# Mixed Convection Flow of Viscoelastic Fluid by a Stretching Cylinder with Heat Transfer

**DOI:** 10.1371/journal.pone.0118815

**Published:** 2015-03-16

**Authors:** Tasawar Hayat, Muhammad Shoaib Anwar, Muhammad Farooq, Ahmad Alsaedi

**Affiliations:** 1 *Department of Mathematics, Quaid-I-Azam University* 45320, *Islamabad* 44000, *Pakistan*; 2 Nonlinear Analysis and Applied Mathematics (NAAM) Research Group, Faculty of Science, King Abdulaziz University P. O. Box 80203, Jeddah 21589, Saudi Arabia; Bascom Palmer Eye Institute, University of Miami School of Medicine;, UNITED STATES

## Abstract

Flow of viscoelastic fluid due to an impermeable stretching cylinder is discussed. Effects of mixed convection and variable thermal conductivity are present. Thermal conductivity is taken temperature dependent. Nonlinear partial differential system is reduced into the nonlinear ordinary differential system. Resulting nonlinear system is computed for the convergent series solutions. Numerical values of skin friction coefficient and Nusselt number are computed and discussed. The results obtained with the current method are in agreement with previous studies using other methods as well as theoretical ideas. Physical interpretation reflecting the contribution of influential parameters in the present flow is presented. It is hoped that present study serves as a stimulus for modeling further stretching flows especially in polymeric and paper production processes.

## Introduction

There is continuous increasing interest of recent researchers in flow problems of non-Newtonian fluids due to their high applications in industry and engineering. It is well known now that the stretched flow problems of non-Newtonian fluids occur in production of plastic, paper and food materials. Heat transfer involvement has great role in these processes. Various recent researchers are engaged in exploring the heat transfer characteristics in the flow of non-Newtonian fluids over a stretching surface. For instance, Turkyilmazoglu and Pop [1] studied exact solution of stagnation point flow of Jeffrey fluid due to stretching/shrinking sheet. Singh and Agarwal [2] presented radiative heat transfer characteristics in the flow of second grade fluid induced by exponentially stretching sheet with porous medium and elastic deformation. Shehzad et al. [3] studied boundary layer flow of Maxwell fluid due to bidirectional stretching surface with prescribed heat flux and prescribed surface temperature. Hayat et al. [4] examined the mixed convection boundary layer flow of Maxwell fluid over a stretching surface in the region of stagnation point with melting effect. Convergent series solutions were constructed by homotopy analysis method. Hayat et al. [5] analyzed radiative flow of third grade fluid with Joule heating. The flow of micropolar fluid over a heated unsteady stretching surface with mixed convection and viscous dissipation is presented by El-Aziz [6]. Mukhopadhyay et al. [7] studied flow of Casson fluid by unsteady stretching surface. Mixed convection viscoelastic fluid flow and heat transfer by permeable stretching surface is analyzed by Turkyilmazoglu [8]. Magnetohydrodynamic mixed convection flow of Powell-Eyring fluid over a nonlinear stretching surface was investigated by Panigrahi et al. [9]. Radiative mixed convection flow of viscoelastic fluid over a porous wedge with magnetic field was examined by Rashidi et al. [10]. Mukhopadhyay and Mandal [11] analyzed heat transfer characteristics in the boundary layer flow of Casson fluid induced by symmetric porous wedge with surface heat flux. Stagnation point mixed convection flow of Maxwell fluid with conjugate heat transfer was presented by Hsiao [12]. Heat and mass transfer in the boundary layer flow of viscoelastic fluid past a stretching surface was studied by Seshadri and Munjam [13]. Dalir [14] examined forced convection flow of Jeffrey fluid over a stretching sheet with entropy generation. Hakeem et al. [15] presented radiative boundary layer flow of walter’s B fluid over a stretching sheet with elastic deformation and non-uniform heat source/sink.

Characteristics of stretching surface in the presence of mixed convection flow have a large number of applications in industry and engineering. The free convection effects gained more importance in the presence of gravitational force. Also phenomena of flow and heat transfer is effected by both stretching and buoyancy forces. Note that thermal buoyancy forces generated due to change in temperature of stretching surface affect the heat transfer rate in manufacturing processes. This phenomenon is pivotal in cooling of electronic devices, solar energy systems, nuclear reactors cooling during emergency shutdown, heat exchangers placed in a low velocity environment, defroster system, boilers, cooling of combustion chamber wall in a gas turbine, automobile demister and flows in the atmosphere and ocean. Patil et al. [16] presented unsteady mixed convection flow over a power law stretching cylinder. Analysis of mixed convection flow over a stretching/shrinking cylinder in the stagnation point region is studied by Lok et al. [17]. Magnetohydrodynamic (MHD) flow of micropolar fluid with slip and mixed convection effects over a vertical shrinking sheet is analyzed by Das [18]. Moradi et al. [19] examined mixed convection-radiation over inclined plate in porous medium. Hayat et al. [20] presented three-dimensional boundary layer mixed convection of viscoelastic fluid with convective boundary condition and thermal radiation. Multivariate weighted complex network analysis for characterizing nonlinaer dynamic behavior in two-phase flow was examined by Gao et al. [21].

Most of the physical properties change with temperature in the real applications. One of those is the variable thermal conductivity which may vary with temperature linearly or non-linearly during the analysis of flow field. For example, in the case of lubricating fluids, the frictional forces increase the temperature of the fluid. As a result rate of heat transfer and thermophysical properties also vary. Mixed convection magnetohydrodynamic (MHD) flow over a stretching sheet with variable viscosity and thermal conductivity is studied by Pal and Mondal [22]. Pal and Chatterjee [23] presented magnetohydrodynamic mixed convection flow of power-law fluid over an inclined plate with Soret and Dufour effect, variable thermal conductivity, chemical reaction, thermal radiation, suction/injection and Ohmic dissipation. Vajravelu et al. [24] analyzed the flow of viscous fluid over a vertical surface with convective boundary conditions and variable fluid properties. Kumar and Sivaraj [25] discussed flow and heat transfer in viscoelastic fluid past a vertical cone and flat plate in the presence of variable properties. Nonlinear complex networks for characterizing oil-gas-water three phase flow was studied by Gao and Jin [26].

Recently the researchers and scientists are interested to reduce the skin friction coefficient and enhance the rate of heating or cooling in the advanced technological processes. Thus various attempts have been made about the reduction of skin friction or drag forces for flows over the surface of a wing, tail plane and wind turbine rotor, etc. However these forces can be reduced by keeping the boundary layer away from separation and to delay the transition of laminar to turbulent flow. This task can be performed through different physical aspects such as moving the surface, through fluid suction and injection and the presence of body forces. Similarly most of the researchers have been tried to enhance the rate of cooling/heating by using different types of boundary conditions over a flat plate. Thus here our main objective is to overcome such difficulties by studying mixed convection flow of second grade fluid over an impermeable stretching cylinder (instead of a stretching flat plate). Effects of variable thermal conductivity and variable surface temperature are considered. Thermal conductivity is assumed to vary linearly with temperature. Section 2 provides the mathematical formulation of the problem. Section 3 comprises methodology i.e., series solutions of the governing equations by homotopy analysis method [27–32]. Results for velocity and temperature for different parameters are discussed in section 4. Conclusion of the present investigations are presented in section 5. Comparison of numerical values of skin friction coefficient and Nusselt number of viscous fluid over a flat plate are also computed and discussed in the limiting cases.

## Mathematical formulation

**Fig 1:**
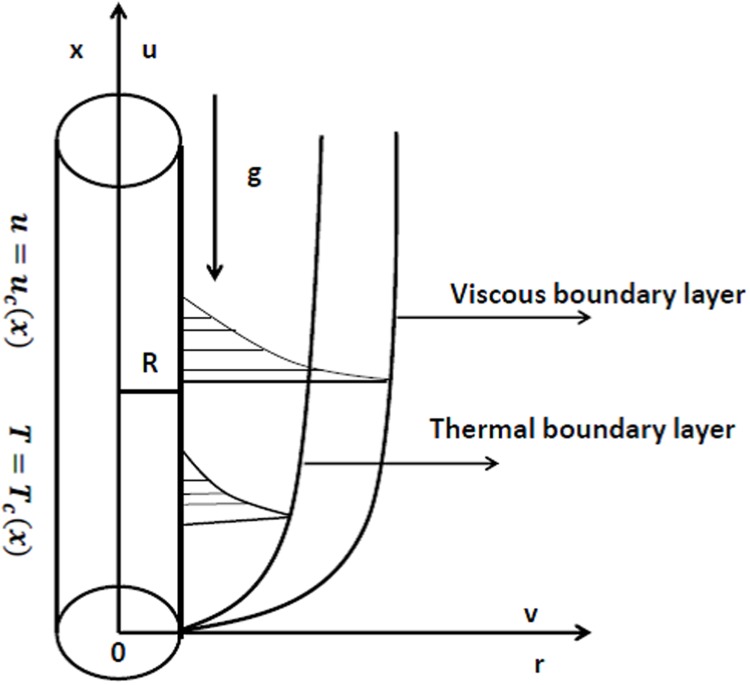
Physical model.

We consider steady incompressible and two-dimensional laminar mixed convection flow of second grade fluid due to a stretching cylinder i.e. stretching velocity *u*_*w*_(*x*) is proportional to axial distance *x* in a linear manner. Boussinesq approximation is used for the buoyancy force (mixed convection). Stretching velocity is produced by applying two equal and opposite forces on the cylinder such that origin is kept constant. The temperature of the cylinder surface *T*_*w*_(*x*) is higher than the ambient fluid temperature *T*_∞_. The cylindrical coordinates are chosen in such a way that *x*-axes is taken along the axial direction of cylinder while *r*-axes is perpendicular to it. Heat transfer analysis is carried out in the presence of variable thermal conductivity. Further thermal conductivity is assumed to vary linearly with temperature. Effect of thermal buoyancy force is considered. The direction of increasing *x*-axis and stretching cylinder is the same since the thermal buoyancy and stretching forces assist each other in the flow field (see Fig. 1). The following equations can govern the present flow consideration:

Equation of continuity

∇.V=0 (1)

Equation of motion

$$\rho \frac{dV}{dt}=\nabla .\tau +\rho b$$ (2)

Energy equation

$$\begin{array}{c} \rho c_{p}\frac{dT}{dt}=\frac{1}{r}\frac{\partial }{\partial r}\left(K\;\left(T\right)\;r\frac{\partial T}{\partial r}\right) \end{array}$$ (3)

where $\frac{d\text{V}}{dt}$ is the material derivative and the Cauchy stress tensor *τ* for second grade fluid is [2]:

$$\begin{array}{c} \tau =-pI+\mu A_{1}+\alpha_{1}A_{2}+\alpha_{2}A_{1}^{2}, \end{array}$$ (4)

**A**_1_ and **A**_2_ represent first and second Rivlin-Ericksen tensors which are defined as follows

$$\begin{array}{c} A_{1}=\text{grad}\;V+{\left(\text{grad}\;V\right)}^{T^{*}} \end{array}$$ (5)

where T* represents transpose

$$\begin{array}{c} A_{2}=\frac{dA_{1}}{dt}+A_{1}\left(\text{grad}\;V\right)+{\left(\text{grad}\;V\right)}^{T^{*}}A_{1} \end{array}$$ (6)

In cylindrical coordinates we have

$$\begin{array}{c} \text{grad}\;V=\left[\begin{array}{ccc} \frac{\partial V_{r}}{\partial r} & \frac{1}{r}\frac{\partial V_{r}}{\partial \theta }-\frac{V_{\theta }}{r} & \frac{\partial V_{r}}{\partial x}\\ \frac{\partial V_{\theta }}{\partial r} & \frac{1}{r}\frac{\partial V_{\theta }}{\partial \theta }+\frac{V_{r}}{r} & \frac{\partial V_{\theta }}{\partial x}\\ \frac{\partial V_{x}}{\partial r} & \frac{1}{r}\frac{\partial V_{x}}{\partial \theta } & \frac{\partial V_{x}}{\partial x} \end{array}\right],\;\;\;\;\nabla .\tau =\left[\begin{array}{c} \frac{\partial \tau_{rr}}{\partial r}+\frac{\tau_{rr}}{r}+\frac{1}{r}\frac{\partial \tau_{\theta r}}{\partial \theta }+\frac{\partial \tau_{xr}}{\partial x}-\frac{\tau_{\theta \theta }}{r}\\ \frac{\partial \tau_{r\theta }}{\partial r}+\frac{\tau_{r\theta }}{r}+\frac{1}{r}\frac{\partial \tau_{\theta \theta }}{\partial \theta }+\frac{\partial \tau_{x\theta }}{\partial x}+\frac{\tau_{\theta r}}{r}\\ \frac{\partial \tau_{rx}}{\partial r}+\frac{\tau_{rx}}{r}+\frac{1}{r}\frac{\partial \tau_{\theta x}}{\partial \theta }+\frac{\partial \tau_{xx}}{\partial x} \end{array}\right] \end{array}$$ (7)

Here *α*_1_ and *α*_2_ are the material constants. For the consistency of model with thermodynamics it is necessary that

$$\begin{array}{c} \alpha_{1}\geq 0,\;\;\;\mu \geq 0,\;\;\;\;\text{and}\;\;\;\;\alpha_{1}+\alpha_{2}=0. \end{array}$$ (8)

Using the velocity field

$$\begin{array}{c} V=[v\;(r,x),0,u\;(r,x)] \end{array}$$ (9)

the governing equations (continuity, momentum and energy equations) after using boundary layer assumptions (i.e. *v* = *O* (*δ*), *r* = *O* (*δ*), *u* = *O* (1) and *x* = *O* (1)) reduce to the forms [33]

$$\begin{array}{c} \frac{\partial (ru)}{\partial x}+\frac{\partial (rv)}{\partial r}=0, \end{array}$$ (10)

$$\begin{array}{ccc} v\frac{\partial u}{\partial r}+u\frac{\partial u}{\partial x} & = & \nu \left(\frac{\partial^{2}u}{\partial r^{2}}+\frac{1}{r}\frac{\partial u}{\partial r}\right)+\frac{\alpha_{1}}{\rho }\left(\begin{array}{c} v\frac{\partial^{3}u}{\partial r^{3}}+u\frac{\partial^{3}u}{\partial x\partial r^{2}}-\frac{\partial^{2}v}{\partial r^{2}}\frac{\partial u}{\partial r}+\frac{\partial u}{\partial x}\frac{\partial^{2}u}{\partial r^{2}}\\ +\frac{1}{r}\left(v\frac{\partial^{2}u}{\partial r^{2}}+u\frac{\partial^{2}u}{\partial x\partial r}-\frac{\partial v}{\partial r}\frac{\partial u}{\partial r}+\frac{\partial u}{\partial x}\frac{\partial u}{\partial r}\right) \end{array}\right)\\ & & +g\beta_{T}\left(T-T_{\infty }\right), \end{array}$$ (11)

$$\begin{array}{c} v\frac{\partial T}{\partial r}+u\frac{\partial T}{\partial x}=\frac{1}{\rho c_{p}r}\frac{\partial }{\partial r}\left(K\;\left(T\right)\;r\frac{\partial T}{\partial r}\right). \end{array}$$ (12)

The relevant boundary conditions are mentioned below:

$$\begin{array}{c} u\left(x,R\right)=u_{c}\left(x\right)=\frac{u_{0}x}{l},\;v\left(x,R\right)=0,\;T\left(x,R\right)=T_{c}\left(x\right)=T_{\infty }+{\left(\frac{x}{l}\right)}^{n}△T,\;\\ u\left(x,r\right)\to 0,\;\;\;\;T\left(x,r\right)\to T_{\infty }\;\;\;\text{as}\;\;\;r\to \infty . \end{array}$$ (13)

In the above expressions *u* and *v* are the velocity components in the *x* and *r* directions respectively, *ν* is the kinematic viscosity, *ρ* is the density, *c*_*p*_ is the specific heat at constant pressure, *u*_*c*_(*x*) is the stretching velocity of the cylinder, *u*_0_ is the reference velocity, *l* is the characteristics length, *n* is surface temperature exponent, *α*_1_ is the second grade material parameter, $△T\left(T_{c}(x)-T_{\infty }={\left(\frac{x}{l}\right)}^{n}△T\right)$ is the characteristics temperature, *T* and *T*_∞_ are the fluid and ambient temperatures respectively, *β*_*T*_ is a coefficient of volumetric thermal expansion. The thermal conductivity *K*(*T*) is given by [22]:

$$\begin{array}{c} K\left(T\right)=K_{\infty }\left(1+\epsilon \theta \right) \end{array}$$ (14)

where *K*_∞_ is the thermal conductivity of the ambient fluid, *θ* is the dimensionless temperature and *ϵ* is a scalar parameter which shows the influence of temperature on variable thermal conductivity. Using the transformations [34]

$$\begin{array}{ccc} \eta & = & \sqrt{\frac{u_{0}}{\nu l}}\;\left(\frac{r^{2}-R^{2}}{2R}\right),\\ u & = & \frac{u_{0}x}{l}f^{\prime }\left(\eta \right),\;\;\;\;\;v=-\frac{R}{r}\sqrt{\frac{u_{0}\nu }{l}}f\left(\eta \right),\;\;\;\theta \left(\eta \right)=\frac{T-T_{\infty }}{T_{c}-T_{\infty }}, \end{array}$$ (15)

equation (10) is identically satisfied and Eqs. (11) and (12) are reduced as follows:

$$\begin{array}{ccc} & & \left(1+2\gamma \eta \right)f\prime \prime \prime +2\gamma f\prime \prime +ff\prime \prime -{\left(f\prime \right)}^{2}+4\gamma \beta \left(f\prime f\prime \prime -ff\prime \prime \prime \right)\\ & & \;+\;\beta \left(1+2\gamma \eta \right)\left(2f\prime f\prime \prime \prime +{\left(f\prime \prime \right)}^{2}-ff\prime \prime \prime \prime \right)+\lambda \theta =0, \end{array}$$ (16)

$$\begin{array}{c} \left(1+2\gamma \eta \right)\;\left(1+\epsilon \theta \right)\;\theta \prime \prime +2\gamma \;\left(1+\epsilon \theta \right)\;\theta \prime +\epsilon \;\left(1+2\gamma \eta \right)\;\theta \prime^{2}+\mathrm{Pr}\left(f\;\theta \prime -nf\prime \theta \right)=0, \end{array}$$ (17)

while boundary conditions reduce to the forms

$$\begin{array}{ccc} f(0) & = & 0,\;\;f\prime \left(0\right)=1,\;\;\;\theta \left(0\right)=1,\;\;\;\;\\ \;f\prime \left(\eta \right) & \to & 0,\;\;\;\;\theta \left(\eta \right)\to 0\;\;\;\;\text{as}\;\;\;\eta \to \infty . \end{array}$$ (18)

In Eqs. 16 and 17 *γ* is the curvature parameter, *λ* is the mixed convection parameter, Pr is the Prandtl number and *β* is the viscoelastic parameter. These parameters are defined as follows:

$$\begin{array}{ccc} \lambda & = & \frac{Gr_{x}}{{\text{Re}}_{x}^{2}}=\frac{g\beta_{T}l^{2}\left(T_{c}-T_{\infty }\right)}{u_{0}^{2}x},\;\;\;\;\;\;\;\;\;\;\;\;\;\;Gr_{x}=\frac{g\beta_{T}\left(T_{c}-T_{\infty }\right)x^{3}}{\nu^{2}},\\ \;\gamma & = & \sqrt{\frac{\nu l}{u_{0}R^{2}}},\;\;\;\;\;\;\;\;\;\;\;\;\;\;\;\;\;\;\;\beta =\frac{\alpha_{1}u_{0}}{\rho \nu l},\;\;\;\;\;\;\;\;\;\;\;\;\;\;\;\;\;\;\;\;\;\;\;\mathrm{Pr}=\frac{\mu c_{p}}{K_{\infty }}, \end{array}$$ (19)

where *Gr*_*x*_ represents the local Grashof number (which is the ratio of buoyancy to viscous forces).

Skin friction coefficient and Nusselt number are defined as follows

$$\begin{array}{c} C_{f}=\frac{2\tau_{c}}{\rho u_{c}^{2}},\;\;\;\;Nu_{x}=\frac{xq_{c}}{k(T_{c}-T_{\infty })} \end{array}$$ (20)

where wall shear stress (*τ*_*c*_) and heat flux (*q*_*c*_) are

$$\begin{array}{c} \tau_{c}={\left(\mu \frac{\partial u}{\partial r}+\alpha_{1}\left(v\frac{\partial^{2}u}{\partial r^{2}}+u\frac{\partial^{2}u}{\partial u\partial r}+\frac{\partial u}{\partial r}\frac{\partial u}{\partial x}-\frac{\partial v}{\partial r}\frac{\partial u}{\partial r}\right)\right)}_{r=R},\;\;q_{c}=-k{\left(\frac{\partial T}{\partial r}\right)}_{r=R}. \end{array}$$ (21)

Nondimensional skin friction coefficient and Nusselt number (which is the ratio of convective to conductive heat transfer coefficients) i.e. local surface heat flux are given by

$$\begin{array}{c} \frac{1}{2}C_{f\;}\sqrt{{\text{Re}}_{x}}=\left(1+3\beta \right)f\prime \prime \left(0\right),\;\;\;\;\;\;\frac{Nu}{\sqrt{{\text{Re}}_{x}}}=-\theta \prime \left(0\right), \end{array}$$ (22)

in which ${\text{Re}}_{x}=\frac{u_{c}(x)x}{\nu }=\frac{u_{0}x^{2}}{\nu l}$ is the local Reynolds number (which is the ratio of inertial to viscous forces).

## Series solutions

Homotopy analysis method was derived from the fundamental concept of topology known as homotopy. Two functions are said to be homotopic if one function can be continuously deformed into the other function. If *f*_1_ and *f*_2_ are two continuous functions which maps from a topological space *X* into topological space *Y* then *f*_1_ is homotopic to *f*_2_ if there exists a continuous map *F*

$$\begin{array}{c} F:\;X\times [0,1]\to Y \end{array}$$ (23)

such that for each *x* ∈ *X*

$$\begin{array}{c} F\left(x,0\right)=f_{1}\left(x\right),\;\;\;\;F\left(x,1\right)=f_{2}\left(x\right) \end{array}$$ (24)

Then map *F* is called homotopic between *f*_1_ and *f*_2_. Homotopy analysis method is proposed by Liao [27] in 1992 which is used to solve the highly nonlinear equations. This method is independent of small or large physical parameters. Homotopy is a continuous deformation or variation of a function or equation. It has several advantages over the other methods i.e., (i) it is independent of small or large parameters (ii) ensure the convergence of series solution (iii) provides great freedom to select the base function and linear operator. Such flexibility and freedom help us in solving the highly nonlinear problems. It is also noted that linear part of the differential equation is selected as the linear operator for the homotopy analysis method. However in semi infinite domain it is preferred in such a way that the solution appears in the form of exponential functions for rapid convergence analysis. Homotopy analysis method requires initial guesses (*f*_0_, *θ*_0_) and linear operators (𝓛_*f*_, 𝓛_*θ*_) in the forms [31]:

$$\begin{array}{c} f_{0}\left(\eta \right)=1-\exp\left(-\eta \right)\;\;\;\;\;\text{and}\;\;\;\;\theta_{0}\left(\eta \right)=\exp\left(-\eta \right), \end{array}$$ (25)

$$\begin{array}{c} {𝓛}_{f}\left(f\right)=\frac{d^{3}f}{d\eta^{3}}+\frac{d^{2}f}{d\eta^{2}}\;\;\;\;\text{and}\;\;\;\;{𝓛}_{\theta }\left(\theta \right)=\frac{d^{2}\theta }{d\eta^{2}}+\frac{d\theta }{d\eta }, \end{array}$$ (26)

with

$$\begin{array}{c} {𝓛}_{f}\left[A_{1}+A_{2}\eta +A_{3}\;\exp\left(-\eta \right)\right]=0,\;\;\;\;\;\;{𝓛}_{\theta }\left[A_{4}+A_{5}\;\exp\left(-\eta \right)\right]=0,\; \end{array}$$ (27)

where *A*_*i*_ (*i* = 1–5) are the arbitrary constants. The zeroth and *m*th order deformation problems are described in the following subsections.

## Zeroth-order problems

Liao [27] constructed a one-parameter family of equations in the embedding parameter *p* ∈ [0, 1] called the zeroth-order deformation equation

$$\begin{array}{c} \left(1-p\right)\;{𝓛}_{f}\;\left[\hat{f}\;\left(\eta ;p\right)-f_{0}\left(\eta \right)\right]=pH_{f}\hbar_{f}{𝒩}_{f}\;\left[\hat{f}\;\left(\eta ;p\right),\;\hat{\theta }\;\left(\eta ;p\right)\right], \end{array}$$ (28)

$$\begin{array}{c} \left(1-p\right)\;{𝓛}_{\theta }\;\left[\hat{\theta }\;\left(\eta ;p\right)-\theta_{0}\left(\eta \right)\right]=pH_{\theta }\hbar_{\theta }{𝒩}_{\theta }\;\left[\hat{\theta }\;\left(\eta ;p\right),\;\hat{f}\;\left(\eta ;p\right)\right], \end{array}$$ (29)

$$\begin{array}{cc} \hat{f}\left(0;p\right) & =0,\;\;\;\;\hat{f}\prime \left(0;p\right)=1\;\;\text{and}\;\;\;\hat{f}\prime \left(\eta ;p\right)\to 0\;\;\text{as}\;\eta \;\to \infty ,\\ & \hat{\theta }\;\left(0;p\right)=1\;\;\;\text{and}\;\;\;\hat{\theta }\;\left(\eta ;p\right)\to 0\;\;\text{as}\;\eta \;\to \infty , \end{array}$$ (30)

$$\begin{array}{cc} {𝒩}_{f}\;\left[\hat{f}\;\left(\eta ;p\right),\hat{\theta }\;\left(\eta ;p\right)\right] & =(1+2\gamma \eta )\;\hat{f}\;\prime \prime \prime +2\gamma \hat{f}\;\prime \prime +\hat{f}\;\hat{f}\;\prime \prime -{\left(\hat{f}\;\prime \right)}^{2}+4\gamma \beta \;\left(\hat{f}\;\prime \hat{f}\;\prime \prime -\hat{f}\;\hat{f}\;\prime \prime \prime \right)\\ & +\;\beta \;\left(1+2\gamma \;\eta \right)\;\left[2\hat{f}\;\prime \hat{f}\;\prime \prime \prime +{\left(\hat{f}\;\prime \prime \right)}^{2}-\hat{f}\;\hat{f}\;\prime \prime \prime \prime \right]+\lambda \hat{\theta }\;, \end{array}$$ (31)

$$\begin{array}{ccc} {𝒩}_{\theta }\left[\hat{\theta }\;\left(\eta ;p\right),\hat{f}\;\left(\eta ;p\right)\right] & = & \left(1+2\gamma \eta \right)\left(1+\epsilon \hat{\theta }\right)\hat{\theta }\;\prime \prime +2\gamma \left(1+\epsilon \hat{\theta }\right)\hat{\theta }\prime \\ & & +\mathrm{Pr}\left(\hat{f}\;\hat{\theta }\prime -n\hat{f}\prime \hat{\theta }\right)+\epsilon \left(1+2\gamma \eta \right){\left(\hat{\theta }\prime \right)}^{2}, \end{array}$$ (32)

Here *p* ∈ [0, 1] is embedding parameter *H*_*f*_, *H*_*θ*_ are the auxiliary functions, ℏ_*f*_, ℏ_*θ*_ are the non-zero auxiliary parameters and $\hat{f}\left(\eta ;p\right)$ and $\hat{\theta }\left(\eta ;p\right)$ are the deformed functions.

## *m*th-order deformation problems

Differentiating the zeroth-order deformation Eqs. (28) and (29) *m*-time with respect to *p*, then dividing by *m*! and finally setting *p* = 0, we get the *m*-th order deformation equations [29]:

$$\begin{array}{c} {𝓛}_{f}\left[f_{m}\left(\eta \right)-\chi_{m}f_{m-1}\left(\eta \right)\right]=\hbar_{f}{𝓡}_{m}^{f}\left(\eta \right), \end{array}$$ (33)

$$\begin{array}{c} {𝓛}_{\theta }\left[\theta_{m}\left(\eta \right)-\chi_{m}\theta_{m-1}\left(\eta \right)\right]=\hbar_{\theta }{𝓡}_{m}^{\theta }\left(\eta \right), \end{array}$$ (34)

$$\begin{array}{c} f_{m}\left(0\right)=0,\;\;\;\;f_{m}^{\prime }\left(0\right)=0\;\;\text{and}\;\;\;f_{m}^{\prime }\left(\eta \right)\to 0\;\;\;\text{as}\;\eta \to \infty ,\\ \theta_{m}\left(0\right)=0\;\;\;\text{and}\;\;\;\theta_{m}\left(\eta \right)\to 0\;\;\;\;\;\text{as}\;\eta \to \infty , \end{array}$$ (35)

$$\begin{array}{l} {𝓡}_{m}^{f}\left(\eta \right)=\left(1+2\gamma \eta \right)f_{m-1}^{\prime \prime \prime }\left(\eta \right)+2\gamma f_{m-1}^{\prime \prime }\left(\eta \right)+\lambda \theta_{m-1}\left(\eta \right)+\sum_{k=0}^{m-1}\left[f_{m-1-k}f_{k}^{\prime \prime }-f_{m-1-k}^{\prime }f_{k}^{\prime }\right]\\ +\sum_{k=0}^{m-1}\left[4\gamma \beta \left(f_{m-1-k}^{\prime }f_{k}^{\prime \prime }-f_{m-1-k}f_{k}^{\prime \prime \prime }\right)+\beta \left(1+2\gamma \eta \right)\left(2f_{m-1-k}^{\prime }f_{k}^{\prime \prime \prime }+f_{m-1-k}^{\prime \prime }f_{k}^{\prime \prime }-f_{m-1-k}f_{k}^{\prime \prime \prime \prime }\right)\right], \end{array}$$ (36)

$$\begin{array}{ccc} {𝓡}_{m}^{\theta }\left(\eta \right) & = & \left(1+2\gamma \eta \right)\theta_{m-1}^{\prime \prime }\left(\eta \right)+\sum_{k=0}^{m-1}2\gamma \epsilon \theta_{m-1-k}^{\prime }\theta_{k}+\sum_{k=0}^{m-1}\mathrm{Pr}\left[f_{m-1-k}\theta_{k}^{\prime }-nf_{m-1-k}^{\prime }\theta_{k}\right]\\ & & +\sum_{k=0}^{m-1}\left[\epsilon \left(1+2\gamma \eta \right)\theta_{m-1-k}^{\prime \prime }\theta_{k}\right]+2\gamma \theta_{m-1}^{\prime }\left(\eta \right)+\sum_{k=0}^{m-1}\left[\epsilon \left(1+2\gamma \eta \right)\theta_{m-1-k}^{\prime }\theta_{k}^{\prime }\right], \end{array}$$ (37)

$$\begin{array}{c} \chi_{m}=\left\{\begin{array}{c} 0,\;\;\;m\leq 1,\\ 1,\;\;\;m>1. \end{array}\right.. \end{array}$$ (38)

For *p* = 0 and *p* = 1 we have

$$\hat{f}\left(\eta ;0\right)=f_{0}\left(\eta \right)\;\;\;,\;\;\hat{f}\left(\eta ;\text{1}\right)\text{=}f\left(\eta \right),$$ (39)

$$\hat{\theta }\left(\eta ;0\right)=\theta_{0}\left(\eta \right)\;\;\;,\;\;\hat{\theta }\left(\eta ;\text{1}\right)\text{=}\theta \left(\eta \right),$$ (40)

Note that when *p* increases from 0 to 1 then $\hat{f}\left(\eta ;p\right)$ and $\hat{\theta }\left(\eta ;p\right)$ vary from the initial solutions *f*_0_(*η*), *θ*_0_(*η*) to the final solutions *f*(*η*), *θ*(*η*) respectively. By Taylor’s series we can write

$$\hat{f}\left(\eta ;p\right)=f_{0}\left(\eta \right)+\sum_{m=1}^{\infty }f_{m}\left(\eta \right)p^{m}\;\;\;\text{with}\;\;\;f_{m}\left(\eta \right)\text{=}{\frac{\text{1}}{m!}\frac{\partial^{m}\hat{f}\left(\eta ;\text{p}\right)}{\partial p^{m}}|}_{p\text{=0}},$$ (41)

$$\hat{\theta }\left(\eta ;p\right)=\theta_{0}\left(\eta \right)+\sum_{m=1}^{\infty }\theta_{m}\left(\eta \right)p^{m}\;\;\;\text{with}\;\;\;\theta_{m}\left(\eta \right)\text{=}{\frac{\text{1}}{m!}\frac{\partial^{m}\hat{\theta }\left(\eta ;\text{p}\right)}{\partial p^{m}}|}_{p\text{=0}},$$ (42)

The value of auxiliary parameter is selected in a proper way so that the series (42) and (43) converge at *p* = 1 i.e.

$$\begin{array}{c} f\left(\eta \right)=f_{0}\left(\eta \right)+\sum_{m=1}^{\infty }f_{m}\left(\eta \right), \end{array}$$ (43)

$$\begin{array}{c} \theta \left(\eta \right)=\theta_{0}\left(\eta \right)+\sum_{m=1}^{\infty }\theta_{m}\left(\eta \right), \end{array}$$ (44)

The general solutions (*f*_*m*_, *θ*_*m*_) of Eqs. (33) and (34) in terms of special solutions $\left(f_{m}^{*},\theta_{m}^{*}\right)$ are given by [28]

$$\begin{array}{c} f_{m}\left(\eta \right)=f_{m}^{⋆}\left(\eta \right)+A_{1}+A_{2}\eta +A_{3}\;\exp\left(-\eta \right), \end{array}$$ (45)

$$\begin{array}{c} \theta_{m}\left(\eta \right)=\theta_{m}^{⋆}\left(\eta \right)+A_{4}+A_{5}\exp\left(-\eta \right), \end{array}$$ (46)

where the constants *A*_*i*_ (*i* = 1–5) are given by

$$\begin{array}{lll} A_{2} & = & -{\left.\frac{\partial f_{m}^{⋆}\left(\eta \right)}{\partial \eta }\right|}_{\eta \to \infty },\;\;\;A_{3}={\left.\frac{\partial f_{m}^{⋆}\left(\eta \right)}{\partial \eta }\right|}_{\eta =0}-{\left.\frac{\partial f_{m}^{⋆}\left(\eta \right)}{\partial \eta }\right|}_{\eta \to \infty },\;\;\;A_{1}=-A_{3}-f_{m}^{⋆}\left(0\right),\\ A_{4} & = & -{\left.\theta_{m}^{⋆}\left(\eta \right)\right|}_{\eta \to \infty }\;\;\;\;\;\text{and}\;\;\;\;\;\;\;\;A_{5}={\left.\theta_{m}^{⋆}\left(\eta \right)\right|}_{\eta \to \infty }-\theta_{m}^{⋆}\left(0\right).\; \end{array}$$ (47)

## Convergence analysis

**Fig 2:**
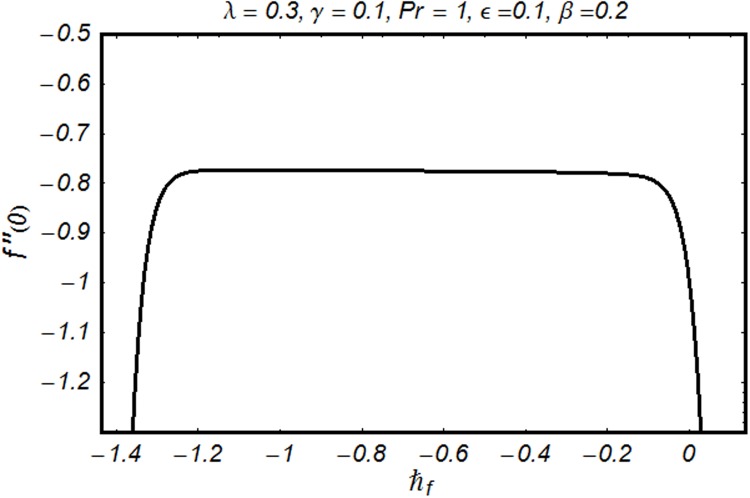
ℏ-curve for *f*.

**Fig 3:**
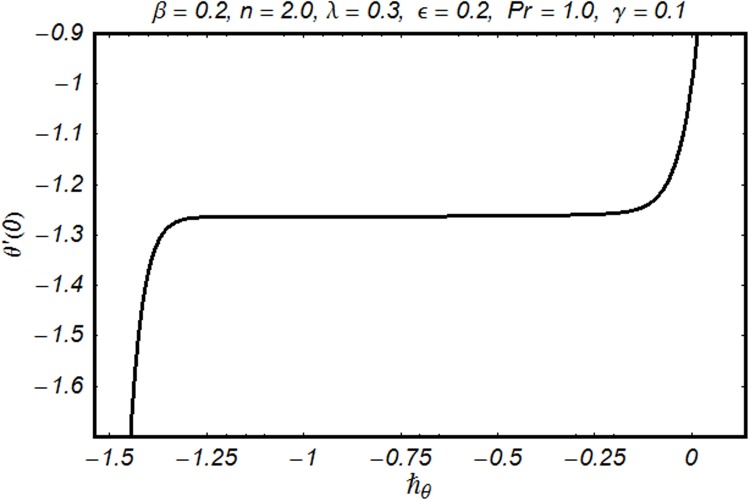
ℏ-curve for *θ*.

To find the series solutions by homotopy analysis method, it is essential to check their convergence. Therefore we have plotted the ℏ-curves in the Figs. 2 and 3. It is seen that permissible values of ℏ_*f*_ and ℏ_*θ*_ are −1.30 ≤ ℏ_*f*_ ≤ −0.15 and −1.25 ≤ ℏ_*θ*_ ≤ −0.35. Convergence of series solution is analyzed through Table 1. It is clear from the table that 32^nd^ and 29^th^ order of approximations are sufficient for *f*″(0) and *θ*′(0) respectively. Comparison of *f*″(0) with earlier results in a limiting case is shown in the Table 2. It is observed that the present results are in good agreement with the previous results. Table 3 examines comparison of *θ*′(0) with the existing work for different values of Pr and *n*. This table reflects agreement for both the results. Influence of various physical parameters on skin friction coefficient and local Nusselt number are shown in Table 4. It is clear from the Table that increase of *γ, β*, Pr and *n* shows that the magnitude of skin friction coefficient increases. However it decreases with the increase of *ϵ* and *λ* keeping all other parameters fixed. Local Nusselt number increases with the increase of *γ, β, λ*, Pr and *n* while it decreases with the increase of *ϵ*.

**Table 1:** Convergence of the series solutions for different order of approximations when *λ* = 0.3, *ϵ* = 0.2, *γ* = 0.1, Pr = 1, *β* = 0.2 and *n* = 2.

Order of approximation	*f*″(0)	*θ*′(0)
1	−0.7971	−1.1654
5	−0.7809	−1.2559
15	−0.7762	−1.2617
25	−0.7746	−1.2629
29	−0.7742	−1.2632
32	−0.7739	−1.2632
35	−0.7739	−1.2632

**Table 2:** Comparison of −*f*″(0) when *γ* = 0, *λ* = 0 and *β* = 0 (i.e., for Newtonian fluid without mixed convection over a flat plate).

Hassanien et al [35]	Andersan et al [36]	Vajravelu et al [37]	Present results
1.0000	1.000	1.00000	1.0000

**Table 3:** Comparison of *θ*′(0) with Hassanien et al. [35] for various values of Pr and temperature exponent *n* when *λ* = 0, *γ* = 0, *β* = 0 and *ϵ* = 0 (i.e., for Newtonian fluid without mixed convection over a flat plate with constant thermal conductivity).

*n*	Pr = 0.72		Pr = 1		Pr = 3	
	Hassanien [35]	Present results	Hassanien [35]	Present results	Hassanien [35]	Present results
−1	0.00000	0.0000	0.00000	0.0000	0.00000	0.0000
0	−0.46325	−0.4698	−0.58198	−0.5825	−1.16525	−1.1654
1	−0.80868	−0.8133	−1.00000	−1.0000	−1.92368	−1.9237

**Table 4:** Effects of various involved parameters on the skin friction coefficient and local Nusselt number.

*γ*	*β*	*λ*	*ϵ*	Pr	*n*	(1 + 3*β*)*f*″ (0)	−*θ*′ (0)
**0**	0.2	0.2	0.2	0.8	2	−1.2187	1.0720
**0.2**						−1.3400	1.1447
**0.4**						−1.4562	1.2160
	**0**					−1.0590	1.1914
	**0.2**					−1.4564	1.2160
	**0.4**					−1.8077	1.2317
		**0**				−1.9902	1.2192
		**0.2**				−1.8109	1.2317
		**0.4**				−1.6858	1.2414
			**0**			−1.7010	1.4034
			**0.2**			−1.6858	1.2414
			**0.4**			−1.6750	1.1251
				**0.5**		−1.6394	0.9139
				**0.8**		−1.6750	1.1251
				**1**		−1.7030	1.2584
					**0**	−1.6108	0.6497
					**1**	−1.6596	0.9895
					**2**	−1.7030	1.2584

## Results

Interpretation of the series solutions with respect to influence of various physical parameters on the velocity and temperature profiles are studied in this section. Graphical analyses of the velocity and temperature fields have been carried out to understand the present mathematical model. All the Figs. are plotted to study the effects of different parameters on the velocity and temperature profiles for temperature exponent *n* = 1 and *n* = 2. Figs. clearly show that fluid velocity *f*′(*η*) and temperature *θ*(*η*) decrease from 1 to 0 as distance from the stretching cylinder increases.

**Fig 4:**
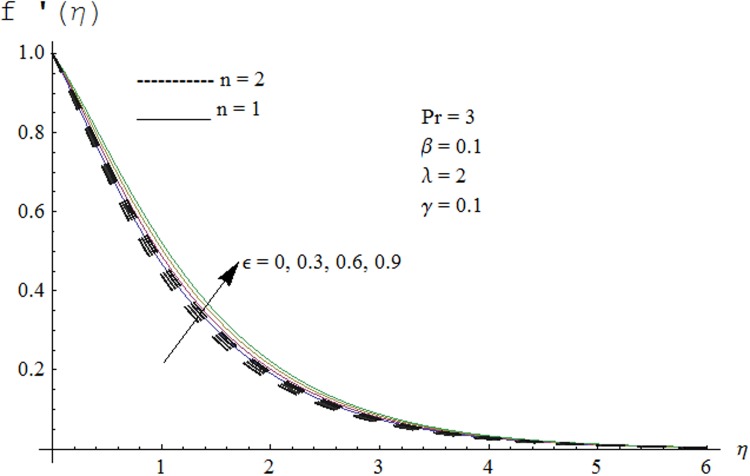
Effect of *ϵ* on velocity profile.

**Fig 5:**
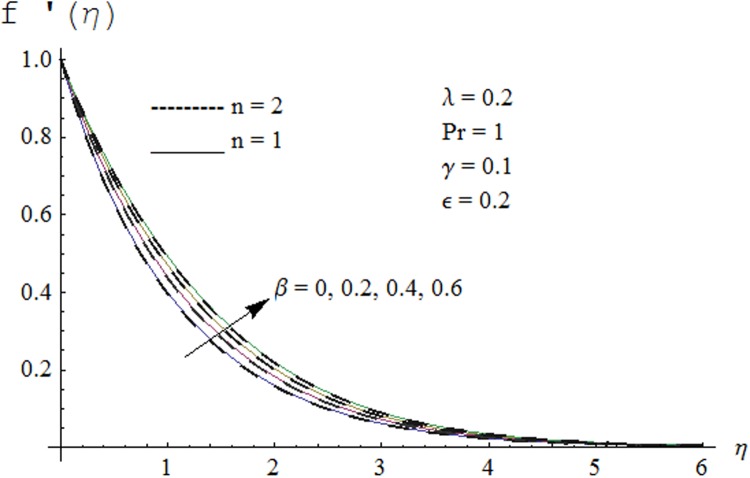
Effect of *β* on velocity profile.

**Fig 6:**
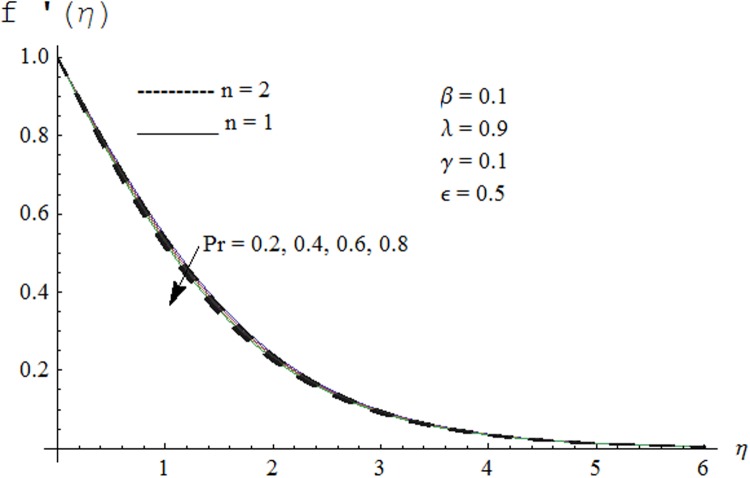
Effect of Pr on velocity profile.

**Fig 7:**
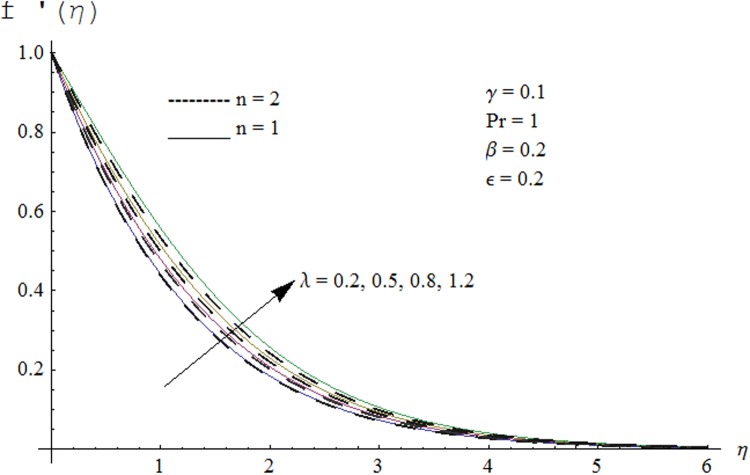
Effect of *λ* on velocity profile.

**Fig 8:**
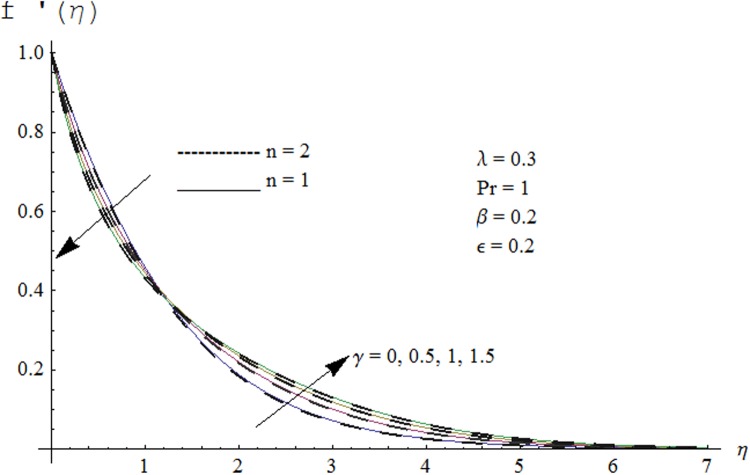
Effect of *γ* on velocity profile.

**Fig 9:**
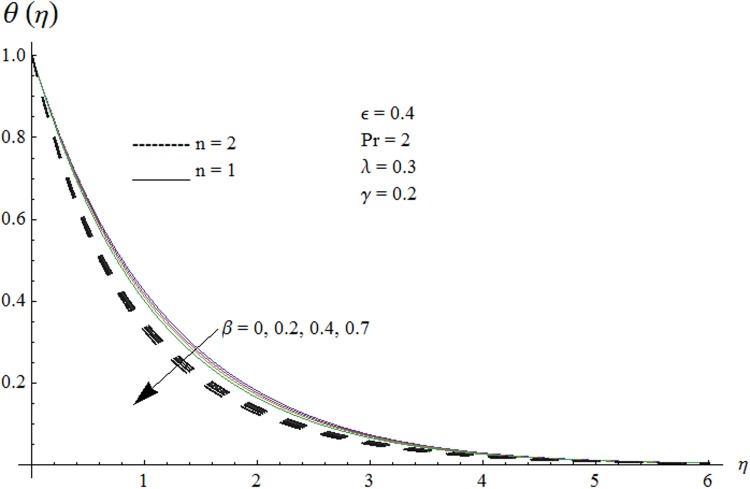
Effect of *β* on temperature profile.

**Fig 10:**
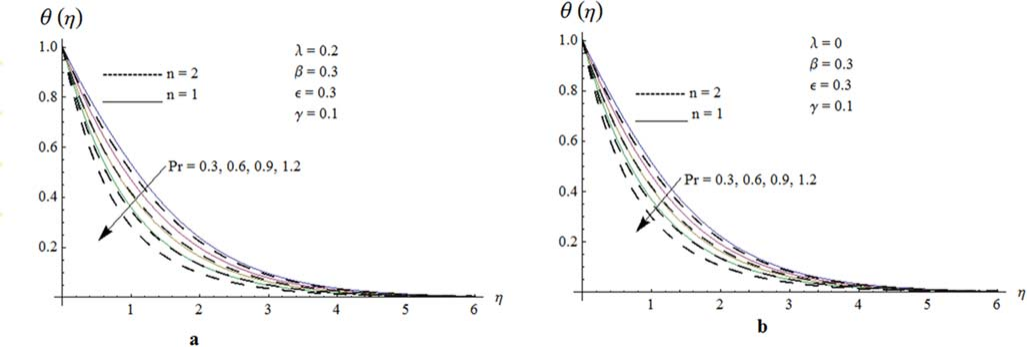
Effect of Pr on temperature profile when *λ* = 0.2 and *λ* = 0.

**Fig 11:**
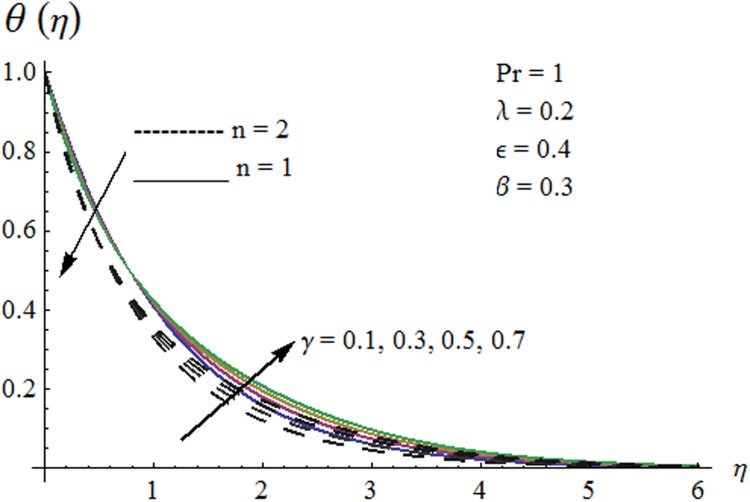
Effect of *γ* on temperature profile.

**Fig 12:**
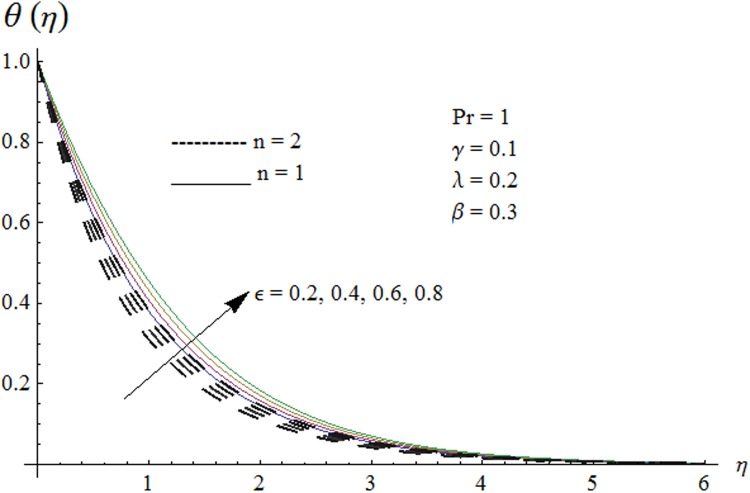
Effect of *ϵ* on temperature profile.

**Fig 13:**
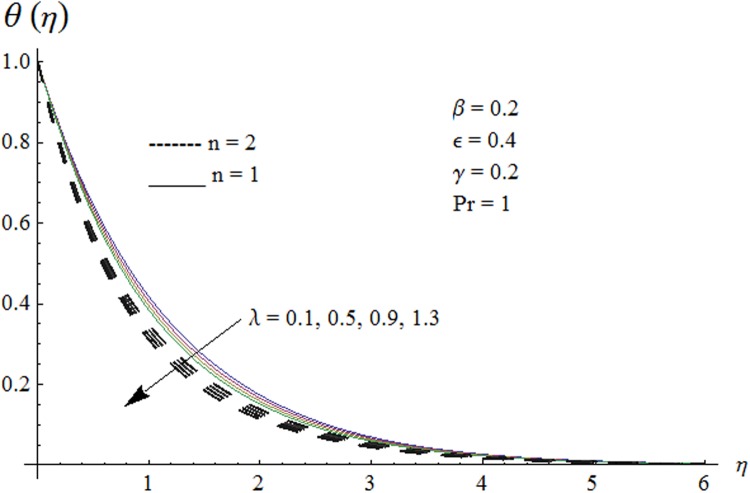
Effect of *λ* on temperature profile.

**Fig 14:**
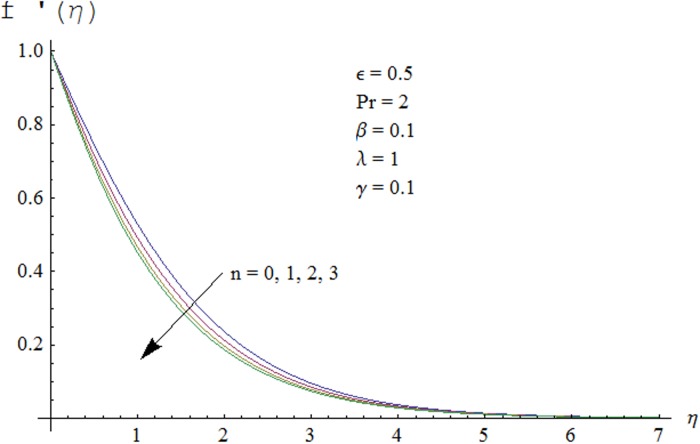
Effect of *n* on velocity profile.

**Fig 15:**
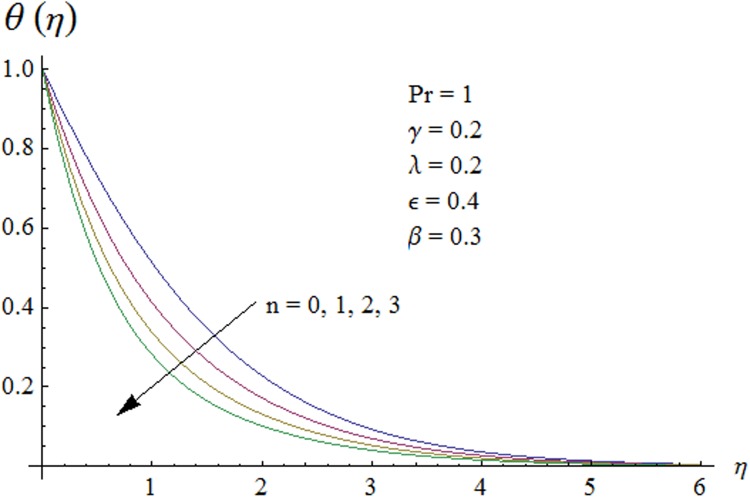
Effect of *n* on temperature profile.

Figs. 2 and 3 are plotted to obtain the values of ℏ_*f*_ and ℏ_*θ*_ for which homotopy analysis solutions remained convergent. It is clear from these Figs. that series solution is convergent at those values of ℏ_*f*_, ℏ_*θ*_ where ℏ-curves for *f, θ* are parallel to ℏ_*f*_ and ℏ_*θ*_ axis respectively [27]. Figs. 4–15 show that the parameters *ϵ, β*, Pr, *λ* and *n* have monotonic variation while *γ* has non-monotonic effects on the velocity and temperature profiles. Characteristic of variable thermal conductivity parameter *ϵ* on the velocity profile is shown in Fig. 4. It is noticed that the velocity profile increases gradually with the increase of *ϵ*. Due to temperature dependent thermal conductivity, viscous boundary layer thickness increases because average thermal conductivity of the fluid increases and consequently magnitude of the velocity profile increases by increasing the value of *ϵ*. Here *ϵ* = 0 and *ϵ* > 0 correspond to the constant and variable thermal conductivity respectively. Fig. 5 is plotted to show the influence of viscoelastic parameter *β* on the velocity profile. Velocity along with associated momentum boundary layer thickness increase when *β* increases. This is consistent with the expression for *β* in equation 19 which shows that *β* increases as the viscosity decreases. So fluid moves easily and as a result velocity profile increases. Fig. 6 is sketched for the influence of Prandtl number on the velocity profile. It is noted that velocity profile decreases with the increase of Pr and momentum boundary layer thickness is at higher level for small values of Pr. Prandtl number is the ratio of momentum diffusivity to thermal diffusivity. With increase in Prandtl number fluid becomes more viscous which results in the reduction of velocity profile. Characteristics of mixed convection parameter *λ* on the velocity profile is displayed in Fig. 7. It is observed that velocity and momentum boundary layer thickness increase for larger *λ*. Mixed convection parameter is the ratio of buoyancy to inertial forces. It is worth mentioning that *λ* = 0 and *λ* ≠ 0 correspond to the absence and presence of mixed convection parameter respectively. Also *λ* > 0 indicates that heat is convected from the surface of cylinder to the fluid flow i.e. cooling of the cylinder surface or heating the fluid. With the increase of mixed convection parameter *λ*, (*T*_*w*_ − *T*_∞_) and buoyancy forces increase. Therefore velocity of the fluid increases. Behavior of curvature parameter *γ* on the velocity profile is illustrated in the Fig. 8. As expected velocity and momentum boundary layer thickness decrease near the surface of cylinder while opposite effects are observed away from it. Because resistance offered in aggregate due to viscous forces near the surface of cylinder is much greater than away from it. Also, with the increase of curvature parameter *γ* radius of cylinder decreases as a result less resistance offered to the fluid motion therefore fluid velocity increases.

Now our attention is to illustrate the temperature for various parameters interest. Therefore effect of *β* on temperature profile is sketched in Fig. 9. It is concluded that temperature along with thermal boundary layer thickness decrease as *β* increases. As mentioned earlier with the increase of *β* viscosity of fluid and friction between fluid layers decrease due to which temperature remains at lower level for higher values of *β*. Influence of Pr on temperature profile is shown in Fig. 10. It is found that temperature and thermal boundary layer thickness decrease with the increase in Pr. Prandtl number is the ratio of momentum diffusivity to thermal diffusivity. With the increase of Prandtl number, thermal diffusivity decreases which results in the reduction of temperature profile. Prandtl number controls the relative thickness of momentum and thermal boundary layers. For larger Pr the heat diffuses slowly and thermal boundary layer becomes thinner when compared to small Pr. Small Pr results in a thicker thermal boundary layer which diffuses heat quickly than the higher Pr. Fig. 11 is plotted to show the influence of *γ* on the temperature profile. It is evident that temperature and thermal boundary layer thickness decrease near the surface and increase away from it. With the increase of curvature parameter rate of heat transfer increases from cylinder to the fluid which is responsible for the reduction of temperature profile near the surface of cylinder while it results in the enhancement of temperature profile away from the surface of cylinder. Influence of *ϵ* on the temperature profile is displayed in Fig. 12. It is clear from the Fig. that temperature profile is higher for large value of *ϵ*. Further thermal boundary layer thickness also increases. As thermal conductivity depends on temperature so thermal boundary layer thickness increases as average thermal conductivity of the fluid increases hence magnitude of temperature profile increases by increasing the value of *ϵ*. Fig. 13 is plotted to show the behavior of mixed convection parameter *λ* on temperature profiles. It is clear from the Fig. that the temperature and thermal boundary layer thickness decrease with the increase of *λ*. With the increase of mixed convection parameter *λ*, rate of heat transfer increases due to high gravitational field. Therefore temperature profile decreases. Influence of temperature exponent *n* on the velocity and temperature profiles is shown in Figs. 14 and 15 respectively. These Figs. depict that both the velocity and temperature along with their respective boundary layer thicknesses decrease as *n* increases. With the increase of temperature exponent *n* difference between wall and ambient temperature increases which corresponds to higher rate of heat transfer as a result temperature as well as velocity decrease with the increase of *n*.

## Concluding remarks

Mixed convection boundary layer flow of second grade fluid by a stretching cylinder is examined. Thermal conductivity is variable. Effects of various parameters are studied graphically as well as in a tabular form. Velocity, temperature profiles and their associated boundary layer thicknesses increase with the increase of variable thermal conductivity parameter. Velocity and temperature profiles increase with the increase of curvature parameter away from the cylinder. Viscoelastic and mixed convection parameters have opposite effects on the velocity and temperature profiles. Velocity and temperature decrease when Prandtl number increases.

It is hoped that present study serves as a stimulus for modeling further stretching flows especially in polymeric, paper production and food processes. The present analysis can be extended for the subclass of rate type fluids explaining relaxation and retardation times phenomena. More third grade fluid case describing shear thickening and shear thinning is also tackled. The analysis can be also seen for the case of variable thermal conductivity in magnetohydrodynamics.
